# The Relationship between Isometric Force-Time Characteristics and Dynamic Performance: A Systematic Review

**DOI:** 10.3390/sports8050063

**Published:** 2020-05-15

**Authors:** Danny Lum, G. Gregory Haff, Tiago M. Barbosa

**Affiliations:** 1 Sport Science and Sport Medicine, Singapore Sport Institute, Singapore 397630, Singapore; 2 Physical Education and Sports Science National Institute of Education, Nanyang Technological University, Singapore 637616, Singapore; barbosa@ipb.pt; 3 Centre for Exercise and Sports Science Research (CESSR), School of Medical and Health Sciences, Edith Cowan University, Joondalup 6027, Australia; g.haff@ecu.edu.au; 4 Directorate of Sports, Exercise and Physiotherapy, University of Salford, Greater Manchester M5 4WT, UK; 5 Department of Sport Sciences, Polytechnic Institute of Bragança, 5300 Bragança, Portugal; 6 Research Centre in Sports, Health and Human Development (CIDESD), 5001 Vila Real, Portugal

**Keywords:** isometric strength test, peak force, rate of force development, impulse

## Abstract

The purpose of this article was to review the data on the relationship between multi-joint isometric strength test (IsoTest) force-time characteristics (peak force, rate of force development and impulse) and dynamic performance that is available in the current literature. Four electronic databases were searched using search terms related to IsoTest. Studies were considered eligible if they were original research studies that investigated the relationships between multi-joint IsoTest and performance of dynamic movements; published in peer-reviewed journals; had participants who were athletes or active individuals who participate in recreational sports or resistance training, with no restriction on sex; and had full text available. A total of 47 studies were selected. These studies showed significant small to large correlations between isometric bench press (IBP) force-time variables and upper body dynamic performances (*r*^2^ = 0.221 to 0.608, *p* < 0.05) and significant small to very large correlation between isometric squat (ISqT) (*r*^2^ = 0.085 to 0.746, *p* < 0.05) and isometric mid-thigh pull (IMTP) (*r*^2^ = 0.120 to 0.941, *p* < 0.05) force-time variables with lower body dynamic performances. IsoTest force-time characteristics were shown to have small to very large correlations with dynamic performances of the upper and lower limbs as well as performance of sporting movements (*r*^2^ = 0.118 to 0.700, *p* < 0.05). These data suggest that IsoTest force-time characteristics provide insights into the force production capability of athletes which give insight into dynamic performance capabilities.

## 1. Introduction

Muscular strength or the ability to produce force against a resistance is an important physical attribute that underpins athletic performance [1,2]. Greater muscular strength is generally associated with a greater muscular power, rate of force development (RFD), and enhanced jumping, sprinting and change of direction performance capacity [1,2]. In addition, strength training interventions have been reported to benefit the performance in a variety of other sports such as endurance running [3,4], swimming [4,5], cycling [4,6] and sprint kayaking [7]. Furthermore, higher level athletes tend to possess greater levels of strength when compared to lower level athletes [8]. Due to its importance, maximum strength is often monitored as part of a performance or monitoring program in order to track training progression. 

The methods that are often used to assess maximal force generating capacity are generally classified as either isometric or dynamic strength assessments [9,10]. Isometric strength testing (IsoTest) requires the athlete to exert force against an immovable device or bar while adopting a specific joint angles or posture such as during the isometric mid-thigh pull (IMTP) (which should replicate the start of the second pull) [11]. The forces generated during these tests are measured with the use of either a strain gauge, cable tensiometer, dynamometer, load cell or a force plate [12,13] allowing for the quantification of peak force (PF) (multi-joint assessment) or peak torque (single-joint assessment). In addition to these measures, IsoTest also allow for the quantification of other force-time characteristics including time specific force values [9,10,14,15,16,17], the rate of force development (RFD) [11,17,18,19,20,21,22] and impulse [22,23,24]. The ability to examine RFD is considered to be an important outcome of IsoTest as this measure is one of the most important physical attributes for the performance of explosive movements [25]. Additionally, isometric impulse has been shown to be significantly related to various dynamic performances such as 1 repetition maximum (1 RM) squat, sprint time and change of direction ability (COD) [22,23]. Therefore, when developing strength assessment test batteries to monitor athletes, the ability to quantify both the RFD and impulse is considered important. 

Isometric strength tests are relatively simple to administer, poses minimal injury risk, have high test-retest reliability, are able to detect subtle changes in strength, and are considered to be less fatiguing than 1RM test [9,11,19,22,26,27,28,29]. Recently there has been an increased interest in exploring the reliability, validity and efficacy of multi-joint IsoTests [26,30]. The PF measured using the IMTP are highly reliable (ICC = 0.89−0.99, CV = 1.7–5%) and is able to determine the smallest detectable change, which appears to be around ~8.5% [26,31]. Additionally, the PF determined during the isometric squat (ISqT) is also highly reliable (ICC = 0.97−0.99, CV = 3.6%) and generally detects the smallest detectable difference in lower limb strength, which appears to be ~11% for ISqT [26,29]. Similarly, the PF achieved during the isometric bench press (IBP) is considered highly reliable (ICC = 0.79−0.98) and is able to determine the smallest detectable change of around ~2% [30,32]. 

In addition to PF measures, there are significant small to very large correlations between several force-time characteristics, such as the RFD and impulse, and performance of sports specific movements in the scientific literature [9,11,22,23,32]. For example, RFD determined during the IMTP exhibits a very large correlation with squat jump (SJ) height (*r =* 0.80, *p* < 0.05) [11]. The isometric impulse across 300 ms has been reported to have a very large inverse correlation with 20-m sprint time (*r* = −0.78, *p* < 0.05) [22]. Due to the relationships between the isometric force-time characteristics and dynamic performances, the quantification of these measures may provide insight into the strength characteristics which underpin these dynamic performances. 

As such there has been an increased interest in using multi-joint IsoTests as a part of performance monitoring programs [17,21,33,34,35,36,37,38,39,40,41,42]. Based upon the current body of scientific knowledge, the force-time characteristics established with multi-joint IsoTests appear to provide valuable information related to an athlete’s ability to execute dynamic sporting movements. However, in order to understand the importance of these measures, sport scientists and practitioners need to recognise how specific force-time characteristics, such as PF, RFD and impulse, determined with IsoTests relate to markers of dynamic sports performance. This understanding can inform decisions about which IsoTest and force-time characteristics need to be included in as part of an assessment battery when designing and administering athlete monitoring programs. 

Therefore, the primary purpose of this article was to review the available data on the relationships between the force-time characteristics (PF, force at various time points, RFD, RFD at various epochs, impulse and impulse at various epochs) determined with multi-joint IsoTests and dynamic performances.

## 2. Materials and Methods

### 2.1. Search Strategy

A systematic search of studies on the relationships between isometric force-time characteristics and dynamic performance was conducted. Original research and review articles were searched and retrieved from electronic searches on Pubmed, SPORTDiscus and Google Scholar databases. PICO search strategy was conducted based on the Boolean technique shown in Table 1. Additional publications were retrieved from the reference lists of the included studies.

### 2.2. Inclusion and Exclusion Criteria

**Figure 1:**
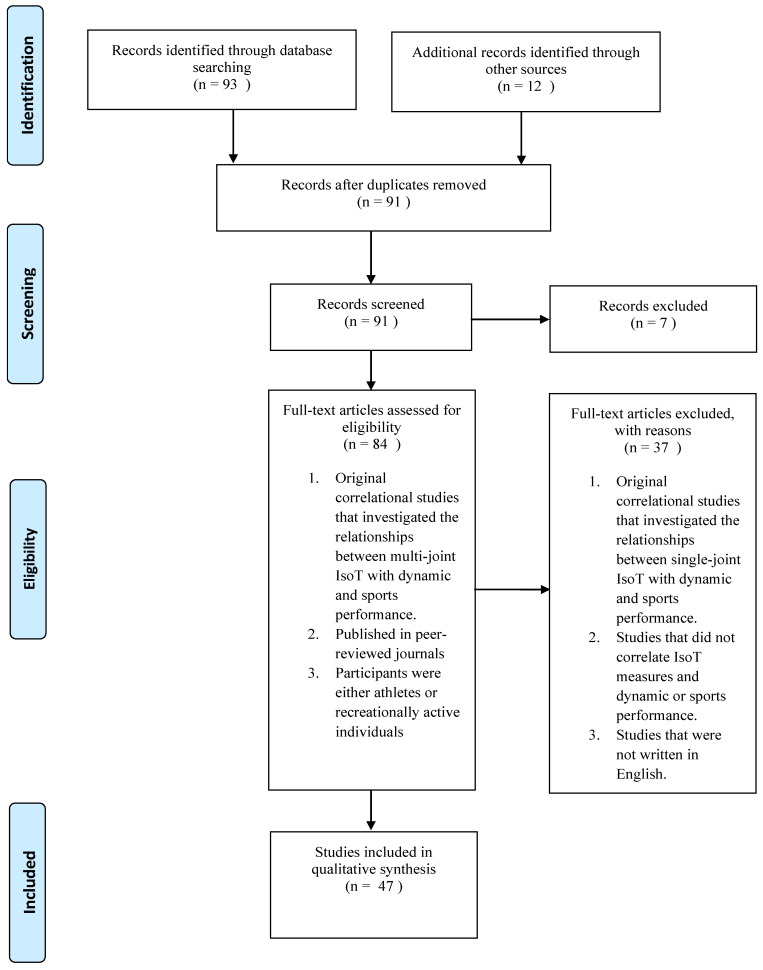
Flow chart detailing the search strategy for the review.

Figure 1 depicts the PRISMA flow diagram identifying, screening, checking eligibility and inclusion of the studies. Studies were included if they: were original research studies that investigated the relationships between multi-joint IsoTests and performance of sports specific movements; published in peer-reviewed journals; had participants who were either athletes or recreationally active individuals who participate in sports or resistance training, with no restriction on sex; and had full text available. Recreationally active individuals were defined as those that were regularly participating in physical activities prior to the studies, but are not participating in competitive sports. Studies were excluded if they: were original research studies that investigated the relationships between single-joint IsoTests and performance of sports specific movements; did not analyze the relationship between isometric force-time characteristics and the performance of sports specific movements; and were not written in English. Search results were screened by two reviewers (DL and TB). There was absence of disagreement during the review process. 

### 2.3. Quality of the Studies

Quality of the studies included was assessed based on the Downs and Black Quality Assessment Checklist [43]. The checklist is used to assess the methodological quality of non-randomized studies. Maximal total score is 27 points, with higher scores indicating better quality. Components of the checklist include (one point per item): (1) Hypothesis/purpose clearly described; (2) Main outcomes are stated in Introduction or Methods; (3) Characteristics of patients were clearly described; (4) Clearly described intervention of interest; (5) Clearly described principal confounders clearly described; (6) Clearly described main findings clearly described; (7) Provided the estimates of random variability for main outcomes; (8) Reported all adverse events of intervention; (9) Described the characteristics of patients who were lost to follow-up; (10) Probability values of main outcomes were reported; (11) Subjects asked to participate were representative of source population; (12) Subjects prepared to participate were representative of source population; (13) Location and delivery of study treatment were representative of source population; (14) Study participants blinded to treatment; (15) Blinded outcome assessment; (16) Clearly described all data dredging; (17) Analyses for differing length of follow-up were adjusted; (18) Appropriate statistical tests performed; 19) Compliance with intervention was reliable; (20) Valid and reliable outcome measures; (21) All recruited participants were from the same population; (22) Recruitment of all participants over the same time period; (23) Randomization of participants to treatment; (24) Investigators and participants were blinded from the allocation of treatment; (25) Adequate adjustment for confounding; (26) Accounted for the losses to follow-up; (27) Sufficient power to detect treatment effect at significance level of 0.05. 

### 2.4. Data Analysis

The first author performed an initial read of the included studies to gain familiarity. Subsequently, each article was re-read and the following information was extracted and inserted in a table to facilitate analysis and presentation: (1) sample size, (2) sex, (3) population, (4) type of isometric assessment, (5) correlation between isometric force-time characteristics and dynamic performance. Where applicable, Fisher r-z transformation was performed to compare the magnitude of correlation between IsoTest measures and dynamic performance reported by different studies. Correlational indices were considered: (i) small, if 0.1 ≤ |*r*| ≤ 0.29; (ii) moderate, if 0.3 < |*r*| ≤ 0.49; (iii) large, if 0.5 ≤ |*r*| ≤ 0.69; (iv) very large, if 0.7 ≤ |*r*| ≤ 0.89; (v) near perfect, if 0.9 ≤ |*r*| ≤ 0.99; and (vi) perfect, if |*r*| = 1 [22].

## 3. Results

One hundred and five publications were obtained from the initial search. After the removal of duplicates (n = 14), publications were filtered by reading the title and abstract, leaving 84 studies that appeared eligible to be included in the present review. A more detailed evaluation of the identified studies resulted in 37 studies being excluded due to their failure to meet the inclusion criteria. The remaining 47 studies were divided into three categories: (1) Relationship between upper limb IsoTests and dynamic measurements (strength and medicine ball throw) (n = 4); (2) Relationship between lower limb IsoTests and dynamic measurements (strength, jump, sprint and COD) (n = 38); and (3) Relationship between IsoTests and performance of sports specific movements (n = 8). Some studies were grouped into more than one category (n = 3).

The quality of the 47 studies were very similar, with Downs and Black Quality Assessment Checklist score ranging from 13 to 14 (Table 2). As the studies included in this systematic review were designed to investigate on the correlation between variables and not designed to investigate on the effects of specific intervention, all studies did not meet the criteria for components 4, 8, 9, 14, 15, 16, 17, 22, 23, 24, 25 and 26. Four studies did not fulfil component 21. 

## 4. Discussion 

### 4.1. Relation Between Upper Limbs Isometric and Dynamic Tests Measurements

The bench press is often used to strengthen the upper body [44] and is often assessed in many sports’ performance testing batteries [7,45]. However, the 1RM bench press test does not provide data on the force-time characteristics for better understanding of an athlete’s force generating capacities. Therefore, it may be important for practitioners to utilise other modes of upper body strength tests in order to allow for a more comprehensive evaluation of an athlete’s neuromuscular capacities. One popular alternative method for assessing upper body strength is the use of the IBP [13,30,32,34,46]. Young et al. [30] have reported that the measurement of IBP PF at multiple elbow angles (ICC = 0.89 to 0.97, %CV = 1.2 to 1.6) were reliable in elite athletes. However, IBP RFD was shown to be unreliable across all elbow angles (ICC = 0.56−0.65, %CV = 0.5−7.6) [30]. One possible reason for the low reliability of RFD could be due to the method used to analyze the data, including filtering and smoothing [10]. These findings indicate that the IBP is a reliable test for measuring PF across the full range of movement but further investigation would be required to ascertain the reliability of RFD. 

As the 1RM bench press is often used for testing upper body strength, various studies have validated IBP against the 1RM bench press and determined the relationship between both tests (Table 3) [12,32,46]. Generally, large to very large relationships have been reported between the PF achieved in the IBP and the 1RM bench press (*r =* 0.570 to 0.78, *p* < 0.05) and RFD (*r =* 0.47, *p* < 0.05). The large variations in these findings may be attributed to the different joint angles used during the IBP in the various studies [46]. For example, Murphy et al. [46] compared the relationship between IBP at a 90° and 120° elbow angle and the 1RM bench press. Only the IBP PF at a 90° elbow angle was significantly correlated with the 1RM bench press. The authors suggested that the best joint angle for an IsoTest should be the joint angle that PF is developed in the dynamic movement of interest. It was suggested that performing the IBP at a 90° elbow angle showed a higher correlation with the 1RM bench press because the sticking point of the bench press movement is close to 90° elbow angle [47]. Hence, the force generated at 90° elbow angle appears to be an important determinant of an athlete’s 1RM bench press. 

Murphy et al. [46] also reported significant relationships between PF achieved in the IBP, performed at 90° elbow angle, and the work done during the bench throw when performed with different loads (*r =* 0.61 to 0.69, *p* < 0.05). The findings of this study were consistent with those reported in previous work by Murphy et al. [32] where there was a significant relationship between the PF achieved in the IBP, when performed at a 90° elbow angle, and work done during bench throw with different loads (*r =* 0.67 to 0.72, *p* < 0.05). However, Murphy and Wilson [48] indicated that the IsoTest had limited value when assessing explosive performance. The authors reported moderate to large correlations between medicine ball throw distance with the PF achieved in the IBP when performed with a 90° (*r =* 0.47, *p* < 0.05) and 120° (*r =* 0.55, *p* < 0.05) elbow angle, but no correlations withpeak RFD achieved at both elbow angles (*r =* 0.08 to 0.31, *p* > 0.05). Similarly, Murphy et al. [32] reported no correlation between IBP PF and peak RFD with seated shot-put distance. The lower correlation between IBP PF and peak RFD with explosive upper body performance may be due to the difference in movement patterns between IBP and the two dynamic movements, or the relatively light load thrown during the medicine ball throw and shot put. Supporting this contention, Murphy et al. [32] reported that the force-time characteristics achieved during an IsoTest are best related to dynamic performances with heavier loads as the PF and RFD resulted in significantly higher correlation with the concentric only bench press at 100% vs. 60% 1RM (PF: *r =* 0.81 vs. 0.69, RFD: *r =* 0.70 vs. 0.48). These observations were also apparent in the study by Murphy et al. [46] where a lower correlation magnitude was reported between IBP and bench press throw when the load was decreased. Collectively these findings suggested that there are strong relationships between the load used during explosive upper body movements and isometric upper body strength force-time characteristics.

### 4.2. Relation Between Lower Limb Isometric and Dynamic Tests Measurements

The importance of possessing high levels of lower body strength is well documented in the scientific literature [2,49]. Moderate to very large correlations exist between lower limb force-time characteristics and dynamic performances such as jump height [15,19,35,50] sprint time and velocity [35,50,51,52,53] and COD test time [22,40,54,55]. Due to these relationships it is important to assess an athlete’s lower body strength in order to monitor the athlete’s training progression and to examine the effectiveness of the training program. 

Two lower limb IsoTests that are useful as part of an athlete monitoring or testing battery are the IMTP and ISqT. The force-time data collected during these tests are strongly correlated to maximum dynamic strength [19,29,37,41,55,56,57] and various markers of dynamic sports performances such as jumping [15,19,35,50], sprinting [35,50,51,52,53,58], throwing [38], boxing [34], golf swing [33], cycling [39] and kayaking [59].

#### 4.2.1. Maximum Dynamic Strength

Multiple studies have investigated the relationship between the 1RM squat with the force-time characteristics achieved from ISqT [23,29,56] and IMTP [21,37,41,53,57,60,61,62]. These studies have reported significant correlation between the 1RM back squat and the PF determined with either the ISqT (*r =* 0.688 to 0.864) [23,29,56] or IMTP (*r =* 0.705 to 0.970) [21,37,41,53,57,60,61,62] (Table 3). These findings suggest that the PF measured during the ISqT and IMTP are closely related to dynamic back squat performance. 

Despite the close relationship between PF measured during the ISqT and IMTP with squat performance, there are several factors that should be considered when substituting the 1RM back squat assessment with either the ISqT or IMTP. One factor to consider is that the PF obtained from ISqT and IMTP are currently not able to be used for planning training loads. The prescription of strength training loads is often based on the 1RM load of the tested exercise and these isometric tests do not provide accurate loads which can be used for exercise prescription. However, Blazevich et al. [56] reported that there was an 8.5% difference to actual 1RM load when the PF produced during ISqT was used to predict the squat 1RM load. Currently, no other known studies have developed a back squat 1RM prediction equation, based on the PF obtained from the ISqT and IMTP, with a low standard error. 

Another factor to consider is that there is a variable degree of relationships between ISqT and IMTP PF and the 1RM squat, which may be explained by difference in the joint angles used when performing ISqT and IMTP. For example, Beckham et al. [63] reported that when IMTP was performed at a 125° knee and a 145° hip angle there are higher forces when compared to performing the test at a 125° knee and a 125° hip angle. Similarly, Palmer et al. [64] reported that PF was higher in ISqT with increasing knee angle. In addition, Bazyler et al. [23] reported that PF during the ISqT performed with a 90° knee angle demonstrated a higher correlation to the 1RM full squat as compared to the PF achieved at 120° knee angle (*r =* 0.864 vs. 0.597). Therefore, variation in the magnitude of the relationship between the PF achieved in the IMTP and ISqT with dynamic performance (e.g. 1RM squat) observed in various studies could be related to the use of different knee and hip angles during the isometric tests [21,23,29,37,41,53,56,57,60,61,62]. (2) The lack of adequate familiarization sessions may exert an effect on the reliability of the data collected. Recently, Drake et al. [29] indicated that a minimum of three familiarization sessions are required to stabilize the learning effect when using the ISqT. Most studies that have investigated the relationships between force-time characteristics obtained from ISqT and the 1RM back squat have not reported any information about the number of familiarizations performed or if any familiarization sessions were performed prior to the ISqT testing [12,21,23,56]. Similarly, studies that have investigated the relationship between IMTP and 1RM back squat did not report the performance of any familiarizations [37,41,53,61]. There is currently no data available to ascertain the number of familiarization sessions required to account for the learning effect of IMTP before measures become stabilized. 

Finally, the third factor to consider is the difference in magnitude of force produced during the IMTP and ISqT. Brady et al. [51] reported that the ISqT PF was significantly higher than the PF achieved in the IMTP by female but not male subjects. Similarly, the RFD (0–200 ms) achieved during the ISqT was significantly higher than RFD (0–200 ms) achieved in the IMTP by female but not male subjects. The authors suggested that the ISqT may be preferred for testing an athletes’ true lower limb maximum force production, especially when working with female athletes.

#### 4.2.2. Jumping

The ability to jump is an important skill that is needed for performing well in many sports [2,8]. The drop jump (DJ), SJ and countermovement jump (CMJ) are often used to assess an athletes’ lower limb power and jumping ability [65,66]. The impact of lower limb strength on jumping ability is well documented in the scientific literature [2,19,34,39,49,67,68]. For example, Berger and Henderson [67] reported that when relatively weak participants focused their training on the development of maximal strength, there were significantly greater increases in lower body power as compared to the changes in performance when power training was the targeted training attribute. Due to the importance of lower body strength and its relationship to jumping performance a number of studies have compared lower body isometric force-time characteristics (i.e. ISqT, IMTP, isometric leg press) and jumping ability (i.e. CMJ, SJ and DJ) in order to determine how these measure relate to one another [1,14,15,16,17,18,19,20,21,22,35,36,39,42,53,57,61,66,69,70].

Significant relationships (*r =* 0.346 to 0.820, *p* > 0.05) have been reported between IMTP force-time characteristics (i.e. absolute and relative PF, RFD and impulse) and force-time characteristics (e.g. height and power) achieved during jumping tasks [11,14,15,17,18,19,21,39,53,58,61,71]. However, careful examination of the available data suggests that there is variability in the relationships between the ISqT force-time characteristics and jump height and power reported in the scientific literature [16,21,34,35,42,57,66,70,71]. For example, Young et al. [70] have reported that there are no significant correlations between the relative PF determined with the ISqT and jump height achieved during the CMJ. Similarly, Nuzzo et al. [21] and Wilson et al. [42] reported that there is no correlation between the absolute and relative PF achieved during the ISqT and jump height, although Nuzzo et al. [21] reported that the absolute PF achieved during the ISqT was significantly correlated with the PF (*r =* 0.639, *p* < 0.05) and peak power (*r =* 0.706, *p* < 0.05) achieved during the CMJ. Young et al. [70] indicated that the lack of relationship between PF determined with the ISqT and jump height achieved during the CMJ may be related to the fact that the ISqT was performed at a knee angle of 120° while the initiation of the concentric phase during the CMJ was initiated from a 90° knee angle. However, Fisher r-z transformation indicated that there is only significant difference between the findings of Markovic and Jaric [66] and Loturco et al. [34] (*p* < 0.05) when the correlation magnitudes between jump height achieved during the CMJ and ISqT performed with a knee angle of 118° [16] and 120° [5,66] were compared to the correlation magnitudes between CMJ height and ISqT performed with a knee angle of 100° [57] and 90° [34,35]. The comparison of findings between these four studies only partially supports the claim by Young et al. [70] that the lack of relationship between ISqT PF and CMJ height was related to the difference in joint angle where force is initiated in both exercises. It is also important to note that the velocity and depth of the countermovement will affect CMJ performance [72]. Therefore, the difference in jump strategy among individuals could have resulted in differences in the relationship between ISqT and CMJ measures.

Although CMJ and SJ assessments are commonly used to measure jump performance and lower body power, the CMJ involves both eccentric and concentric muscle actions while the SJ involves only a concentric muscle action. Therefore, the relationship between the performance (i.e. height and power) of these activities and isometric force-time characteristics may differ. However, a non-significant difference in a Fisher r-z transformation analysis (*p* > 0.05) indicates that the magnitude of correlation between the CMJ height and the PF achieved during IMTP (*r =* 0.346 to 0.820) and ISqT (*r =* 0.480 to 0.790) were similar to the magnitude of correlation between SJ height and PF during IMTP (*r =* 0.400 to 0.870) and ISqT (*r =* 0.790) [14,15,21,34,35,39,66,70]. Similarly, the magnitude of the correlation between the SJ height and the RFD achieved during IMTP (*r =* 0.480) and ISqT (*r =* 0.800) were similar to the magnitude of correlation between CMJ height and the RFD during IMTP (*r =* 0.430 to 0.570) and ISqT (*r =* 0.760) [11,15,53]. 

Haff et al. [11] reported that the RFD during IMTP was significantly correlated with SJ height (*r =* 0.820, *p* < 0.05) but not CMJ height (*r =* 0.070, *p* > 0.05). In contrast, Kraska et al. [15] reported that there were similar correlations between the RFD during IMTP and the jump heights achieved during both the SJ (*r =* 0.480, *p* < 0.05) and CMJ (*r =* 0.430, *p* < 0.05) tests. The difference in findings between Haff et al. [11] and Kraska et al. [15] could be due the difference in the method for calculating RFD [10]. Another reason could be due to the training background of the participants. Haff et al. [11] examined these relationships with weightlifters, while Kraska et al. [15] utilised athletes from multiple sports. To get more insights about the relationships between lower body IsoTest force-time characteristics with CMJ and SJ performance, further studies comparing homogenous and heterogenous population appears to be required. 

Currently, only Nuzzo et al. [21] have investigated the relationship between the relative and absolute PF achieved during the ISqT and IMTP in order to determine the relationship between isometric force-time characteristics and vertical jump performance capacities. Significant correlations were reported between relative PF achieved in IMTP and jump height achieved during the CMJ (*r =* 0.588, *p* < 0.05), but there were no correlations reported between absolute PF achieved in IMTP and CMJ height (*r =* 0.276, *p* > 0.05). These findings supported the results reported by studies that showed no relationship between relative PF achieved from IMTP and jump height achieved from CMJ [17,20], but was in conflict with studies that have reported significant moderate to very large correlations (*r =* 0.346 to 0.82) between these two measurements [14,15,19,39,53]. Discrepancy in the scientific literature may be related to the differences in joint angles adopted during the execution of IMTP [63]. For example, IMTP was performed at 140° knee angle (hip angle was not reported) in the study by Nuzzo et al. [21] while IMTP in the study by Kraska et al. [15] was performed at 120–135° knee angle and 170–175° hip angle. As such, it is important for researchers to standardize the method of performing IMTP. Readers can refer to Comfort et al. [73] for more information on the standardization and methodological considerations for the IMTP.

#### 4.2.3. Sprinting

Sprinting is another fundamental skill required for successful performance in many sports [2,9]. The ability to sprint consists of two phases, the acceleration and maximum speed phases, which have been related to specific physical abilities [74]. Studies examining the relationship between IsoTests, sprint acceleration and maximal speed performance have reported significant relationships between both the ISqT and IMTP force time characteristics and sprint acceleration performances in different athletes [16,17,22,35,40,51,53,74].

Townsend et al. [53] reported that the average velocity achieved during a 20-m sprint was significantly correlated with the PF (*r =* 0.704, *p* < 0.01) and RFD achieved between 0 − 200 ms and 0 – 250 ms (*r =* 0485 to 0.493, *p* < 0.05) during an IMTP test. Additionally, peak velocity achieved during the 20-m sprint was significantly correlated to PF achieved during the IMTP (*r =* 0.536, *p* < 0.01). In addition, Thomas et al. [22] reported that 5 and 20-m sprint times were correlated to PF (*r =* −0.570 to −0.690, *p* < 0.05), maximum RFD (*r =* −0.580 to −0.710, *p* < 0.05), impulse at 100 (*r =* −0.710 to −0.750, *p* < 0.01) and 300 ms (*r =* −0.740 to −0.780, *p* < 0.01) measured with IMTP in collegiate soccer and rugby players. 

Tillin et al. [16] revealed that ISqT force at 100 ms was significantly correlated with 5-m (*r =* −0.630, *p* < 0.01) and 20-m (*r =* −0.540, *p* < 0.05) sprint time in both rugby athletes and untrained adults. Furthermore, Lum and Joseph [35] reported that significant correlationships between ISqT PF (*r =* −0.420, *p* < 0.05), peak RFD (*r =* −0.570, *p* < 0.05) and RFD 0−90 ms (*r =* −0.550, *p* < 0.05) with 20 m sprint time. Altogether, these studies have shown that both ISqT and IMTP were able to provide indication of athletes’ force generating capacities that relate to sprint acceleration. 

The aforementioned study that showed significant relationship between force-time characteristics obtained from both IMTP and ISqT with sprint acceleration performance had used male participants [16,17,22,35,40,51,53,74]. Interestingly, Brady et al. [51] showed no relationship between force-time characteristics obtained from both IMTP and ISqT with sprint acceleration performance in female sprint athletes. The authors attributed this finding to the low sample size for female subjects (n = 10). Further investigation will be required to determine the relationship between isometric force-time characteristics with sprint acceleration performance in female population.

In contrast to sprint acceleration, the relationship between force-time characteristics achieved from IMTP and ISqT and maximum sprint velocity has not been investigated. Therefore, our current knowledge of the relationships between IsoTest force-time characteristics and sprint performance are still confined to the acceleration phase. Future studies should aim to investigate the relationships between IsoTest force-time characteristics and sprint performance over a longer distance.

#### 4.2.4. Change of Direction Ability

Similar to sprinting, the ability to execute rapid CODs are an important ability for successful performance in many sports [75]. Studies have reported significant inverse relationships between COD test time with 1RM back squat [55] and front squat [76]. Additionally, Spiteri et al. [77] have reported that athletes with higher lower body eccentric and isometric strength were able to perform better during the T-Test and 505 COD test. The authors suggested that greater eccentric strength allowed stronger athletes to apply greater braking forces, which led to an increased propulsion force during the COD movement. Additionally, greater isometric force production capacity may allow for optimization of triple extension of the lower body as it enables the maintenance of the lower limbs position during the braking and propulsive phase of the movement. As such, athletes are able control the shift in body position and transfer force towards a new direction, resulting in a faster COD movement [77]. This explanation of how isometric strength contributes to COD movement has been supported by Spiteri et al. [55] who reported very large correlations between the IMTP relative PF with T-test time (*r =* −0.854, *p* < 0.001). The authors suggested that the T-test involved a number of directional changes, which puts demand on an athletes’ ability to control their body orientation. Theoretically, greater isometric force production capacity allows athletes to better control their body and effectively transfer force to a new direction, it is logical that PF achieved during the IMTP resulted in a high magnitude of correlation with T-test time. The relationship between COD ability and force-time characteristics achieved from IMTP has also been investigated in other studies [22,40,41,53,55]. 

Thomas et al. [22] showed that IMTP PF was significantly correlated to performance in the modified 505 test (*r =* −0.570, *p* < 0.05). This finding was comparable to that observed in the study by Hori et al. (2008) where significant relationships between the 1RM hang power clean and front squat with performance in the modified 505 test (*r =* −0.410, *p* < 0.05 and *r =* −0.510, *p* < 0.05, respectively) were reported. In addition, Townsend et al. [53] and Wang et al. [41] found significant inverse correlation between IMTP PF (*r =* −0.657, *p* < 0.01) and RFD (peak & 30 – 250 ms) (*r =* −518 to −0.528, *p* < 0.05) with pro agility test performance. The findings from the studies on relationship between IMTP force-time characteristics and COD ability agree with the results presented by Hori et al. [76] and Nimphius et al. [78]. Therefore, the IMTP may be considered a useful tool for understanding the strength characteristics that underpin COD ability in athletes.

### 4.3. Relationship Between Isometric Tests and Dynamic Sports Performance 

Several studies have investigated the relationship between IsoTest force-time characteristics and dynamic sports performance measures such as: kayaking [59,79,80] shot put [38], boxing [34], cycling [39] and golf [33,81] (Table 4). These studies have suggested that IsoTest force-time characteristics are correlated to the respective sports performance measures.

#### 4.3.1. Sprint Kayaking 

The importance of muscular strength and power on sprint kayak performance has been well documented in the scientific literature [7,59,79,80,82]. These studies have used dynamic strength tests such as the 1RM pull up and the 1RM bench press, and an IsoTest that simulates the pulling phase of a kayak stroke in order to compare the relationship between upper body strength and sprint kayak performance. The 1RM pull up and bench press were shown to have large to very large inverse correlation with the 200 m, 500 m and 1000 m times (*r =* −0.590 to −0.790, *p* < 0.01), in male and female kayakers [7]. 

The relationship between isometric PF with 200-m kayaking time has also been studied [59,79,80]. For example, van Someran & Palmer [80] measured isometric PF using a dynamometer positioned in a way that allowed the athletes to simulate a kayak stroke, simultaneously performing trunk rotation, shoulder extension and elbow flexion, exerting a pulling force in the horizontal plane. Results from the study showed that isometric PF was not significantly correlated with 200-m kayaking time (*r =* −0.370, *p* > 0.05). In contrast, van Someran and Howatson [59] reported significant correlation between isometric PF and 200-m (*r =* −0.470, *p* < 0.05) and 500-m (*r =* −0.600, *p* < 0.01) times in another group of kayak athletes using the same IsoTest as van Someran and Palmer [80]. 

The findings of van Someran and Howatson [59] agreed with that of Uali et al. [79]. In this study, participants performed an isometric bilateral bench pull and isometric one arm cable row [79]. For the isometric bilateral bench pull, subjects assumed a prone position on a high bench and pulled on a fixed barbell maximum force. Similar to the isometric test in the study by van Someran and Palmer [80], the isometric cable row was performed in a position that simulated a kayak stroke. Large to very large correlations between isometric bilateral bench pull PF and isometric one arm cable row PF with kayaking time to 2 m, 5 m and 10 m (*r =* −0.718 to −0.801, *p* < 0.01 and *r =* −0.643 to −0.731, *p* < 0.05, respectively) and peak velocity (*r =* 0.834, *p* < 0.01 and *r =* 0.637 to 0.653, *p* < 0.01, respectively) were reported. Furthermore, the results in this study also showed that the magnitude of correlation between isometric bilateral bent pull force-time characteristics and kayak performance measures were similar to that of the 1RM bilateral bent pull and time to 2 m, 5 m and 10 m (*r =* −0.755 −0.811, *p* < 0.05) and peak velocity (*r =* 0.838, *p* < 0.01). This suggests that PF achieved during the IsoTest used in the study by Uali et al. [79] can provide specific insights into the strength required for kayaking performance. 

Currently, two out of three studies in the literature report that IsoTest force-time characteristics are correlated with kayak performance. However, only van Someran and Howatson [59] have reported moderate to large correlations between IsoTest PF and kayak performances over two full race distances (200 and 500 m). Uali et al [79] reported large to very large correlations between PFs achieved during the isometric bilateral bench pull and isometric one arm cable row with the acceleration phase. In addition, although McKean and Burkett [7] report large correlations between 1RM bench press and kayak performance times over various distances, the relationship between the IBP and kayak performance has not been investigated. Furthermore, no study has compared the relationship between lower body isometric strength force-time characteristics and kayak performance although it has been reported that force produced by the lower limb muscles contributed to 21% of mean paddle stroke force and 16% of mean kayak speed [83]. Based on these findings, it would be worth investigating the relationships between IMTP force-time characteristics and kayaking performance as PF achieved from IMTP has been shown to have near perfect correlation to not only 1RM squat, but to 1RM bench press (*r =* 0.99, *p* < 0.05) as well [37]. Further studies are required to verify this as findings from these studies could provide information on the relation of force production of upper and lower limbs at different joint angles to kayaking performance. 

#### 4.3.2. Shot Put and Bag Throw

Performance during throwing events, such as the shot-put, can be improved by increasing maximal strength [84]. Therefore, it is beneficial to monitor an athlete’s progression using various strength tests in order to evaluate the overall effectiveness of their training program. Stone et al. [38] investigated the relationship between shot put and weight throw distance with IMTP force time characteristics over an 8 weeks strength training period. Both performances were assessed pre-training and 4- and 8-weeks post-training. The IMTP PF was significantly correlated to shot put (*r =* 0.670 to 0.750, *p* < 0.05) and weight throw (*r =* 0.700 to 0.790, *p* < 0.05) distances, with the magnitude of correlation increasing over the training period. These findings suggest that the PF achieved during an IMTP test can provide significant insights into an athlete’s lower body strength levels and how this strength relates to shot put and weight throw performances. Therefore, IMTP testing has been suggested to be a viable option for monitoring the training progression of shot-put athletes [38]. However, more studies investigating the relationship between IMTP and shot-put performance with athletes from different competitive levels are required to expand upon the current body of scientific knowledge [38]. In addition, the PF achieved during the IMTP may also provide insights into the relationship of strength with other throwing performances, such as overhead throwing. This contention is based upon the work of Freeston et al. [85] who report that CMJ height, which is typically correlated with IMTP PF [19,20,21,39,53], showed large correlation (*r =* 0.510, *p* < 0.05) with overhead throw distance. While the connection between the PF achieved during the IMTP is logical, more research is necessary to confirm the hypothesis that there is a relationship between IMTP PF and overhead throwing performance.

#### 4.3.3. Boxing

Successful boxing performance is characterized by the ability to land point scoring punches on the opponents or deliver a punch that knocks out the opponent. These punches are delivered at different speeds and varying degrees of force [86]. Although the punching action involves movement of the arm, trunk, and legs, contribution from the lower limbs to an effective punch was higher than that from both upper limbs and trunk in senior ranked boxers [87]. This information suggests that multi-joint strength tests are more suitable when assessing a boxer’s strength levels. 

A recent case study detailing the physiological profile of a professional boxer during the preparation period reports that there is a reduction in punch impact force throughout the preparatory training period which is accompanied by a concomitant decrease in the PF achieved in both the IMTP and IBP [88]. Additionally, Loturco et al. [34] verified the relationships between punching impact forces and mechanical variables measured during the ISqT, CMJ, SJ, IBP, dynamic bench press and bench throw. These results indicated that the jab and cross at a fixed position, along with the jab and cross at a self-selected position have large to very large correlation with the PF achieved during the ISqT (*r =* 0.680 to 0.830, *p* < 0.01). The same boxing performance markers were also shown to be significantly correlated with height achieved during SJ (*r =* 0.670 to 0.780, *p* < 0.01) and CMJ (*r =* 0.670 to 0.800, *p* < 0.01). The authors stated that the impact forces that are generated during punching are the direct result of the summation of forces applied simultaneously by the upper and lower body. In order for a boxer to produce high impact forces during a punch, he or she must have the ability to transfer the momentum of force from the lower body to the upper body. This may partially explain the high correlations that can be found between punching impact forces and force-time characteristics of lower body strength and jump height. 

Although ISqT PF is correlated to punching impact force, the PF achieved during the IBP was not significantly correlated with punching impact forces. One possible explanation why the ISqT PF was highly correlated to punching impact forces and the PF achieved during the IBP was not may be related to the biomechanics of the punching movement [34]. The lower body movement starts from a zero velocity, which requires the boxer to have the ability to apply a high amount of force against the ground to accelerate the body. Conversely, the extension of the arms occurs at high speed due to the sequential extension of the knee and hip followed by trunk rotation leading into arm extension. This explanation appears to be consistent with the findings of Cabral et al. [89] who reported that the twisting motion of the trunk start in the lower body and proceed distally to the pelvis, trunk and arms. Based on these two studies, it is logical to hypothesize that core muscle strength affects impact forces during punching. This suggestion is supported by Lee and McGill [90] who reported that training the core muscles increased punching impact forces. 

Findings from the study by Loturco et al. [34] have also shown that lower body isometric force production measured using an ISqT is able to provide insights into the amount of punching impact force a boxer can produce. Future studies should investigate the combined relationship between force-time characteristics from IsoTest for core muscles and lower limbs with punching impact force. 

#### 4.3.4. Cycling

Cycling peak power and economy has been shown to benefit from both dynamic and isometric strength training [4,6,91,92,93]. For example, the study by Beattie et al. [91] reported that there was a 10.4% increase in strength measured using IMTP after a period of strength training, was accompanied by an 8.4% improvement in peak power during a 6 s sprint test. Additionally, there was an 8.5% and 4.9% increase in absolute and relative power output at maximal oxygen consumption, respectively. These findings suggest that muscular strength is an important physical attribute contributing to cycling performance. 

Stone et al. [39] investigated the relationship between IMTP force time characteristics, CMJ and SJ performances and cycling performance. The IMTP PF was significantly correlated with both Wingate peak power (*r =* 0.740, *p* < 0.05) and track cycling split times (*r =* −0.490 to −0.550, *p* < 0.05). Although the magnitude of correlation between IMTP PF and track cycling split times were slightly lower to those shown between the jump peak power and split times (*r =* −0.560 to −0.690, *p* < 0.05). Fisher r-z transformations indicate that there was no significant difference between relationship IMTP, CMJ and SJ with cycling split times (*p* > 0.05). These data suggest that the IMTP is able to measure a cyclist’s lower body strength in a way that can be directly related to cycling performance. Future studies should aim to compare the relationships between IMTP force and RFD at various epochs with cycling performance measures (time and power output) in cyclists of different competitive levels.

#### 4.3.5. Golf

The ability to express strength and power with the lower body is considered an important factor that influences the club head velocity during a golf swing [94,95]. For example, Keogh et al. [96] reported that club head velocity during a golf swing is significantly correlated with lower body strength (*r =* 0.533, *p* < 0.05) as measured using the 1RM Hack squat. Additionally, Leary et al. [33] have reported that there are relationships between IMTP force-time characteristics and club head velocity. Specifically, allometrically scaled force at 150 ms approached a significant level of correlation with the average (*r =* 0.46, *p* = 0.07) and maximum (*r =* 0.47, *p* = 0.06) club head velocity. The lack of relationship between IMTP PF and club head velocity found in this study may be related to the difference in time taken to reach PF during the IMTP (1550 ms) and time required to achieve peak ground reaction force during a golf swing (200−300 ms). The authors suggested that the ability to apply forces with the lower body in approximately 150 – 200 ms is an important ability to produce high club head velocity. 

In agreement with the suggestion made by Leary et al. [33], Wells et al. [81] reported significant correlation between club head velocity and IMTP RFD from 0–150 ms (*r =* 0.343, *p* < 0.05) and RFD from 0–200 ms (*r =* 0.398, *p* < 0.05). However, in contrast to the findings of Leary et al. [33], Wells et al. [81] showed significant correlations between IMTP PF and club head velocity (*r =* 0.482, *p* < 0.01). The conflicting results between these two studies may be related to the difference in skill proficiency of that athletes tested. The participants in the study by Wells et al. [81] had a handicap level of 2.7 ± 1.9, while those in the Leary et al. [33]’s study had a handicap level of 14.5 ± 7.3. Additionally, Nesbit and Serrano [97] provided evidence that higher skilled golfers (handicap level closer to 0) work at slower rates during the start of the downswing when compared to golfers with lower skill levels (handicap level above 10). This may have allowed golfers with higher skill levels to generate greater amounts of force prior to hitting the ball (>284 ms). This may partially explain why the IMTP PF correlated to clubhead velocity in the study by Wells et al. [81]. 

Results from the study by Wells et al. [81] suggest that coaches can use IMTP to measure the force development or RFD at different time frames to monitor the effectiveness of the physical training for golfers of various skill levels, as Leary et al. [33] suggested that rapid force development is an important factor in developing high golf club speed. As IMTP PF was only shown to have correlation with golf club speed in golfers of high skill levels (handicap levels ≤ -5) [81], it may be possible that when IMTP PF increases there would be potential to have an increase in club head speed in golfers of this competitive level only.

As discussed in this review, IsoTest force-time characteristics such as PF, RFD and impulse have been shown to be correlated to various performances of sports specific movements. This indicates that IsoTest force-time characteristics were able to provide insights into the athlete’s strength performance capacities that appear to underpin performances of these movements. Therefore, practitioners should consider using IsoTest such as IMTP and ISqT to monitor athletes’ strength progression. Available data on the relationship between IsoTest force-time characteristics and sports performance in the current literature are limited to sprint kayaking, cycling, boxing, throwing and golf. Future studies should investigate on the relationship between IsoTest and other sports performances to add on to the body of knowledge.

### 4.4. Gaps in the Research

There are several research gaps in the current literature on IsoTest. Firstly, although IsoTest such as IMTP, ISqT and IBP have been studied, to the authors’ knowledge no IsoTest for upper body pulling strength has been validated against a 1RM strength test of the same movement. Secondly, studies on IsoTest have only investigated the relationship between force-time variables and performance in a small number of sports. Data on the relationship between IsoTest force-time characteristics and popular sports such as swimming and endurance running, which have been shown to benefit from increased muscular strength, is still not available in the current literature. Thirdly, although motor unit recruitment patterns are different for dynamic and isometric performances, IsoTest force-time characteristics have been shown to result in moderate to high correlation with dynamic performances. The underlying physiological mechanism for this phenomenon remains unclear. Finally, although this review attempt to systematically review the relationship between isometric force-time characteristics and dynamic performances, a meta-analysis was not performed. A meta-analysis might be able to allow the results of available data to be generalized to a larger population, and improve on the precision and accuracy of estimates. Therefore, future studies should aim to address these issues.

## 5. Conclusions

IsoTest are relatively simple to administer, pose minimal risk of injury, have high test-retest reliability, and are able to detect subtle changes in strength that dynamic tests may not be sensitive enough to detect. This mode of strength testing also allows for the analysis of force-time characteristics such as RFD, impulse and force epoch, to be obtained giving a more comprehensive description of an athlete’s force generation capacities. It has been shown that IsoTest force-time characteristics are moderately to very strongly correlated to dynamic performances of the upper and lower limbs as well as performance of sports specific movements, indicating that isometric force-time characteristics were able to provide insights to the force production capability of an athlete for performing the movements discussed in this review. It is important to note that that the best joint angle at which IsoTest should be performed at, may be the joint angle at which PF is developed in the dynamic movement of interest (with the exception of IMTP, which is supposed to mimic the second pull of the clean movement). With this in mind, practitioners can adopt this method to monitor the training progression of their athletes and evaluate the effectiveness of the training program.

## Figures and Tables

**Table 1:** PICO process and Boolean search technique.

Population	Intervention	Comparison (i.e., Design)	Outcome
Human* Subject* Athlete* Participant* Male* Female*	Isometric test Isometric mid-thigh pull Isometric squat Isometric bench press	Cross sectional design Correlation	Sports performance Dynamic performance Peak force Rate of force development Impulse Impact Jumping Sprinting Cycling Rowing Kayaking Swimming

* Truncation.

**Table 2:** Characteristics of studies included in systematic review.

Authors	n	Sex	Population	Downs and Black Quality Assessment Checklist Score
Bailey et al (2013)	36	M	Collegiate athletes	14
Baker et al. (1994)	22	M	Weight trained adults	14
Bazyler et al. (2015)	17	M	Resistance trained adults	14
Beattie et al. (2017)	45	M	Collegiate & recreational athletes	14
Beckham et al. (2013)	12	F & M	Weightlifters	14
Berger & Henderson (1996)	66	M	Physical Education Students	14
Blazevich et al. (2002)	14	M	Competitive & recreational athletes	13
Brady et al. (2019)	25	F & M	Sprint athletes	14
Dos’ Santos et al. (2017)	43	F & M	Sports athletes	13
Drake et al. (2018)	42	M	Strength trained adults	14
Haff et al. (2005)	6	F	Elite weightlifters	14
Haff et al. (1997)	8	M	Weightlifters	14
Kawamori et al. (2006)	8	M	Collegiate weightlifters	14
Khamoui et al. (2011)	19	M	Recreational athletes	14
Kraska et al. (2009)	63	F & M	Collegiate athletes	14
Kuki et al (2017).	25	M	Collegiate soccer players	14
				14
Leary et al. (2012)	12	M	Recreational golfers	14
Loturco et al. (2016)	15	F & M	Amateur boxers	14
Lum & Joseph (2019)	24	F & M	Elite floorball athletes	14
Marcora & Miller (2000)	14	M	Resistance trained adults	14
Markovic & Jaric (2007)	159	M	Physical Education Students	14
McGuigan et al. (2010)	8	M	Collegiate wrestlers	14
McGuigan et al. (2006)	26	M	Resistance trained adults	14
McGuigan & Winchester (2008)	21	M	Collegiate football players	14
Murphy & Wilson (1996)	24	M	Physically active adults	14
Murphy et al. (1994)	13	M	Weight trained adults	14
Murphy et al. (1995)	13	M	Weight trained adults	14
Nuzzo et al. (2008)	12	M	Collegiate athletes	14
Spiteri et al. (2014)	12	F	Elite basketball athletes	14
Stone et al. (2003)	11	F & M	Collegiate Throwers	14
Stone et al. (2004)	30 20	MF & M	Cyclist Cyclist	14
Thomas et al. (2017)	26	F	Netball players	14
Thomas et al. (2015)	14	M	Collegiate athletes	14
Thomas et al. (2015)	22	M	Collegiate athletes	14
Tillin et al. (2013)	26	M	Rugby athletes and untrained adults	13
Townsend et al. (2017)	23	F & M	Collegiate athletes	14
Uali et al. (2012)	10	F & M	Elite junior kayakers	14
van Someran & Howatson (2008)	18	M	Competitive kayakers	14
van Someran & Palmer (2003)	26	M	International & national level kayakers	13
Wang et al. (2016)	15	M	Collegiate rugby athletes	14
West et al. (2011)	39	M	Professional rugby players	14
Wells et al. (2018)	27	M	Golfers	14
Wilson et al. (1995)	15	M	Competitive sports athletes	14
Young & Bilby (1993)	18	M	College students	14
Young et al. (1995)	20	F & M	Junior track and field athletes	14
Young et al. (1999)	29	M	Active adults	14

F = Female, M = Male.

**Table 3:** Relation between upper and lower limbs isometric and dynamic tests measurements.

Authors	Type of Dynamic Performance	Tests Studied	Results
Bailey et al (2013)	Lower limb	IMTP; SJ and CMJ with and without 20kg external load.	IMTP peak force symmetry index vs. - SJ height (*r =* -0.39 to -0.52) - CMJ height (*r =* 0.47 to -0.49) - SJ peak power (*r =* 0.34 to 0.43) - CMJ peak power (*r =* 0.28 to 0.34)
Baker et al. (1994)	Upper limb	1RM bench press; bench press.	IBP peak force vs. - 1RM bench press (*r =* 0.614).
Bazyler et al. (2015)	Lower Limb	ISqT at 90° and 120° knee angles; 1RM full squat and partial squat (100° knee angle).	ISqT 90° peak force vs - 1RM full squat (*r =* 0.864) - 1RM partial squat (*r =* 0.705) ISqT 90° impulse vs - 1RM full squat (*r =* 0.697) - 1RM partial squat (*r =* 0.726) ISqT 90° RFD vs - 1RM full squat (*r =* 0.554) ISqT 120° peak force vs - 1RM full squat (*r =* 0.597) - 1RM partial squat (*r =* 0.789) ISqT 120° impulse vs - 1RM full squat (*r =* 0.575) - 1RM partial squat (*r =* 0.616) ISqT 120° RFD vs - 1RM full squat (*r =* 0.427) - 1RM partial squat (*r =* 0.423)
Beattie et al. (2017)	Lower limb	IMTP; DJ from 30, 40, 50 & 60 cm.	IMTP peak force vs. - DJ height from 30 cm (*r =* 0.429), 40 cm (*r =* 0.364); 50 cm (*r =* 0.404), 60 cm (*r =* 0.481) - RSI from 30 cm (*r =* 0.302), 50 cm (*r =* 0.327), 60 cm (*r =* 0.349). IMTP relative peak force vs - DJ height from 50 cm (*r =* 0.0.338), 60 cm (*r =* 0.443) - RSI from 40 cm (*r =* 0.304); 50 cm (*r =* 0.360), 60 cm (*r =* 0.425).
Beckham et al. (2013)	Lower limb	IMTP; Maximum snatch, and clean and jerk.	IMTP peak force vs. - Snatch (*r =* 0.830) - Clean and jerk (*r =* 0.838) - Total snatch, clean and jerk (*r =* 0.838). IMTP force at 100, 150, 200 and 250 ms vs. - Snatch (*r =* 0.646, 0.636, 0.732 & 0.801, respectively) - Clean and jerk (*r =* 0.643, 0.605, 0.714 & 0.801, respectively) - Total snatch, clean and jerk (*r =* 0.647, 0.621, 0.724, 0.804, respectively). IMTP RFD 0-250 N.s^−1^ vs. - Snatch (*r =* 0.645) - Total snatch, clean and jerk (*r =* 0.603) IMTP RFD 0-250 N.s^−1^ vs. - Snatch (*r =* 0.781) - Clean and jerk (*r =* 0.722) - Total snatch, clean and jerk (*r =* 0.751).
Berger & Henderson (1996)	Lower limb	ISqT; dynamic squat; vertical jump.	ISqT peak force vs. - Vertical jump power (*r =* 0.64).
Blazevich et al. (2002)	Lower limb	ISqT at 90° knee angle and forward hack squat at 90° hip angle and 110° knee angle; 1RM squat and forward hack squat.	ISqT peak force vs. - 1RM squat (*r =* 0.77). Isometric forward hack squat vs. - 1RM forward hack squat (*r =* 0.76).
Brady et al. (2019)	Lower limb	IMTP; ISqT at 90-150° knee angle; 30 m sprint	IMTP peak force vs. - 5 m sprint time (*r =* -0.626). IMTP force at 100, 150 and 200 ms vs. - 5 m sprint time (*r =* -0.585, -0.616 and -0.611, respectively). IMTP RFD at 0-150 and 0-200 ms vs. - 5 m sprint time (r = -0.550 and -0.556, respectively). ISqT peak force vs. - 5 m sprint time (*r =* -0.714). ISqT force at 100, 150 and 200 ms vs. - 5 m sprint time (r = -0.547, -0.589 and -0.541, respectively). ISqT RFD at 0-150 and 0-200 ms vs. - 5 m sprint time (*r =* -0.575 and -0.521, respectively).
Dos’ Santos et al. (2017)	Lower limb	IMTP; 1RM power clean; CMJ; SJ.	IMTP peak force vs. - RSImod (*r =* 0.389); - 1RM power clean (*r =* 0.674). IMTP force at 100ms vs. - 1RM power clean (*r =* 0.633). IMTP force at 150 and 200ms vs. - RSImod (*r =* 0.426 & 0.449, respectively) - 1RM power clean (*r =* 0.569 & 0.629, respectively). IMTP force at 250 ms vs. - CMJ height (*r =* 0.346) - RSImod (*r =* 0.426 & 0.449) - 1RM power clean (*r =* 0.569 & 0.629).
Drake et al. (2018)	Lower limb	ISqT at 90° knee angle; 1RM squat at 90° knee angle	ISqT peak force vs. - 1RM load (*r =* 0.688) - Relative 1RM load (*r =* 0.759)
Haff et al. (2005)	Lower limb	IMTP; DMTP at 30% isometric peak force and 100kg; Maximum snatch and clean and jerk; CMJ; SJ.	IMTP peak force vs. - CMJ peak power (*r =* 0.88) - SJ peak power (*r =* 0.92) - DMTP 30% peak force (*r =* 0.96) - DMTP 100kg peak power, peak force and velocity (*r =* 0.93, 0.99, 0.80) - Maximum snatch (*r =* 0.93) - Maximum total snatch, clean and jerk (*r =* 0.80). IMTP peak RFD vs. - Peak power of CMJ (*r =* 0.81) - SJ (*r =* 0.84) - Maximum total snatch, clean and jerk (*r =* 0.80).
Haff et al. (1997)	Lower limb	IMTP; Dynamic midthigh pull; SJ; CMJ.	IMTP peak force vs. - Dynamic midthigh pull peak force (*r =* 0.80) - SJ peak force (*r =* 0.76). IMTP RFD vs. - Dynamic midthigh pull RFD (*r =* 0.84) - SJ peak power (*r =* 0.76) and height (*r =* 0.80).
Kawamori et al. (2006)	Lower limb	IMTP; DMTP; SJ; CMJ	IMTP peak force vs. - Heavy DMTP peak force (*r =* 0.82) and RFD (*r =* 0.69 to 0.74) - CMJ peak force (*r =* 0.87), RFD (*r =* 0.85), peak power (0.95) and height (0.82) - SJ height (*r =* 0.87).
Khamoui et al. (2011)	Lower limb	IMTP; Dynamic high pull at 30% peak isometric force; Vertical jump with arm swing.	IMTP relative peak force vs. - Vertical jump height (*r =* 0.61) and peak velocity (*r =* 0.62) - Dynamic high pull peak velocity (*r =* -0.61). IMTP RFD at 50 and 100 ms vs - Dynamic high pull peak velocity (*r =* 0.56 & 0.56, respectively) - Dynamic high pull rate of velocity development (*r =* 0.52 & 0.49, respectively).
Kraska et al. (2009)	Lower limb	IMTP; Weighted (20 kg) & unweighted SJ; Weighted (20 kg) & unweighted CMJ.	IMTP peak force vs. - Weighted & unweighted SJ height (*r =* 0.55, 0.40, respectively) - Weighted & unweighted CMJ height (*r =* 0.55, 0.36, respectively). IMTP force at 50 ms vs. - Weighted & unweighted SJ height (*r =* 0.52, 0.47, respectively) - Weighted & unweighted CMJ height (*r =* 0.52, 0.41, respectively). IMTP force at 90 ms vs. - Weighted SJ height (*r =* 0.37) - Weighted CMJ height (*r =* 0.33). IMTP force at 250 ms vs. - Weighted & unweighted SJ height (*r =* 0.56 & 0.39, respectively) - Weighted & unweighted CMJ height (*r =* 0.54 & 0.34, respectively). IMTP RFD vs. - Weighted & unweighted SJ height (*r =* 0.66 & 0.48, respectively) - Weighted & unweighted CMJ height (*r =* 0.62 & 0.43, respectively). All IMTP variables vs. - percent drop in jump height from weighted to unweighted SJ (*r =* -0.24 to -0.43) - percent drop in jump height from weighted to unweighted CMJ (*r =* -0.3 to -0.51).
Kuki et al. (2017)	Lower limb	IMTP; CMJ; DJ; 30-m Sprint (flying start).	IMTP force at 100 ms vs. - DJ index (*r =* 0.433) - Sprint time at 10 m, 30 m and 20-30 m (*r =* -0.521, -0.417 & -0.444, respectively).
Loturco et al. (2016)	Lower limb	ISqT & bench press; SJ; CMJ.	ISqT peak force vs. - SJ height (*r =* 0.79) - CMJ height (*r =* 0.79). ISqT RFD vs. - SJ Height (*r =* 0.80) - CMJ height (*r =* 0.76).
Lum & Joseph (2019)	Lower limb	ISqT; 20 m sprint; CMJ	ISqT peak force vs. - 5 and 20 m sprint times (*r =* -0.42 and -0.42, respectively) - CMJ height (*r =* 0.43 to 0.56). ISqT peak RFD vs. - 5, 10 and 20 m sprint times (*r =* -0.52 to -0.6, -0.52 to -0.61, -0.52 to -0.63, respectively) - CMJ height (*r =* 0.56 to 0.71). ISqT RFD (0-90 ms) vs. - 5, 10 and 20 m sprint times (*r =* -0.53 to -0.63, -0.51 to -0.66, -0.52 to -0.66, respectively) - CMJ height (*r =* 0.61 to 0.68).
Marcora & Miller (2000)	Lower limb	Isometric leg press at 90° and 120° knee angles; CMJ; SJ.	Isometric peak force at 120° vs. - CMJ height (*r =* 0.5) - SJ height (*r =* 0.53) Isometric RFD at 120° vs. - CMJ height (*r =* 0.69) - SJ height (*r =* 0.71)
Markovic & Jaric (2007)	Lower limb	ISqT at 120° knee angle; hopping in place; SJ; CMJ; DJ; 1RM squat; weighted SJ.	ISqT peak force vs. - SJ power & height (*r =* 0.35 & 0.54, respectively) - CMJ power & height (*r =* 0.34 & 0.39, respectively) - DJ power & height (*r =* 0.29 & 0.24, respectively) - Weighted SJ force (*r =* 0.39) - 1RM squat (*r =* 0.38).
McGuigan et al. (2010)	Lower limb	IMTP; 1RM power clean, squat and bench press; Vertical jump	IMTP peak force vs - 1RM power clean (*r =* 0.97) - 1RM squat (*r =* 0.96). IMTP RFD vs. - Coaches ranking of wrestlers (*r =* 0.62).
McGuigan et al. (2006)	Lower limb	IMTP; 1RM squat and bench press; Vertical jump.	IMTP peak force vs. - 1RM squat (*r =* 0.97) - 1RM bench press (*r =* 0.99) - Vertical jump (*r =* 0.72).
McGuigan & Winchester (2008)	Lower limb	IMTP; 1RM squat, power clean & bench press; 2RM split jerk; CMJ; SBJ.	IMTP peak force vs. - 2RM split jerk (*r =* 0.72) - Body mass (*r =* 0.53) - All 1RM measures (*r =* 0.61-0.72).
Murphy & Wilson (1996)	Upper limb	IBP at 90° and 120° elbow angles; Seated medicine ball throw.	IBP peak force at 90° vs. - Seated medicine ball throw distance (*r =* 0.47) IBP peak force at 120° vs. - Seated medicine ball throw distance (*r =* 0.55).
Murphy et al. (1994)	Upper limb	IBP at 90° elbow angles; 1RM bench press; bench press throws at 10 kg and 30% 1RM; concentric only bench press at 30%, 60% and 100% 1RM; eccentric only bench press at 100%, 130% and 150% 1RM; seated shot put.	IBP peak force vs - 1RM bench press (*r =* 0.78), - Bench throw at 10 kg (*r =* 0.72) - 30% 1RM (*r =* 0.67), - Concentric only bench press at 60% (*r =* 0.69) and 100% (*r =* 0.81) 1RM - Eccentric only bench press at 100% (*r =* 0.69), 130% (*r =* 0.55) and 150% (*r =* 0.65) 1RM. IBP RFD vs. - Bench throw at 10 kg (*r =* 0.68) - 30% 1RM (*r =* 0.63) - Concentric only bench press at 100% (*r =* 0.70) 1RM - Eccentric only bench press at 150% (*r =* 0.69) 1RM.
Murphy et al. (1995)	Upper limb	IBP at 90° and 120° elbow angles; 1RM bench press; bench press throws at 15%, 30% and 60% 1RM.	IBP peak force at 90° vs. - 1RM bench press (*r =* 0.78) - Bench press throw 15% (*r =* 0.61), 30% (*r =* 0.69) and 60% 1RM (*r =* 0.67). IBP RFD vs. - Bench press throw 60% 1RM (*r =* 0.59).
Nuzzo et al. (2008)	Lower limb	ISqT and IMTP at 140° knee angle; 1RM squat and power clean; CMJ.	ISqT peak force vs - 1RM squat (*r =* 0.624) - CMJ peak force (*r =* 0.639) and peak power (*r =* 0.706) ISqT RFD vs. - CMJ peak force (*r =* 0.721) and peak power (*r =* 0.776) IMTP peak force vs. - 1RM power clean (*r =* 0.74) - CMJ peak power (*r =* 0.75). Relative IMTP peak force vs. - CMJ height (*r =* 0.588) IMTP RFD vs. - CMJ peak power (*r =* 0.653)
Spiteri et al. (2014)	Lower limb	IMTP; 1RM squat; CMJ; 505 COD; T-test; Agility test.	Relative IMTP peak force vs. - 505 COD (*r =* -0.792) - T-test (*r =* 0.854)
Stone et al. (2003)	Lower limb	IMTP; Dynamic midthigh pull; Snatch.	IMTP peak force vs. - Dynamic midthigh at 30% & 60% peak force (*r =* 0.77 to 0.88, 0.85 to 0.92, respectively) - Dynamic midthigh at 30% & 60% peak power (*r =* 0.77 to 0.81, 0.60 to 0.87, respectively) - Snatch (*r =* 0.94 to 0.98)
Stone et al. (2004)	Lower limb	Part 1 & 2: IMTP; CMJ; SJ.	IMTP peak force vs. - CMJ height (*r =* 0.59 to 0.67) and peak power (*r =* 0.79 to 0.85) - SJ height (*r =* 0.51 to 0.66) and peak power (*r =* 0.78 to 0.86) - Wingate peak power (*r =* 0.74 to 0.90) - Track cycling split times (*r =* -0.49 to -0.55)
Thomas et al. (2017)	Lower limb	IMTP; SJ; CMJ; 10-m sprint; 505 COD.	IMTP peak force vs. - 505 COD (*r =* -0.48 to -0.66) - 5-m sprint time (*r =* -0.49)
Thomas et al. (2015)	Lower limb	IMTP; 20-m sprint; Modified 505 COD.	IMTP peak force vs. - 5 & 20 m sprint time (*r =* -0.57 to -0.69) - Modified 505 COD (*r =* -0.57) IMTP RFD vs. - 5 & 20 m sprint time (*r =* -0.58 to -0.71) - Modified 505 COD (*r =* -0.57). IMTP impulse at 100 ms vs. - 5 & 20 m sprint time (*r* = -0.71 to -0.75) - Modified 505 COD (*r* = -0.58). IMTP impulse at 300 ms vs. - 5 & 20 m sprint time (*r* = -0.74 to -0.78) - Modified 505 COD (*r* = -0.62).
Thomas et al. (2015)	Lower limb	IMTP; CMJ; SJ.	IMTP peak force vs. - CMJ peak force (*r =* 0.45) - SJ peak power (*r =* 0.46) IMTP impulse at various time point vs. - CMJ peak force (*r =* 0.43 to 0.64) and peak power (*r =* 0.43 to 0.51) - SJ peak force (*r =* 0.50 to 0.58) and peak power (*r =* 0.59 to 0.60)
Tillin et al. (2013)	Lower limb	ISqT and explosive squat at ~118° knee angle; 20-m sprint; CMJ.	ISqT peak force vs - CMJ height (*r =* 0.48). ISqT force at 100,150, 200, 250 ms vs. - CMJ height (*r =* 0.51, 0.61, 0.57 and 0.51, respectively) ISqT force at 100 ms vs. - Sprint time (5 & 20 m) (*r =* -0.5). Normalised ISqT force at 100 ms vs. - 5 and 20m sprint time (*r =* -0.42 and -0.54, respectively).
Townsend et al. (2017)	Lower limb	1RM front squat and hang clean; IMTP; CMJ; Proagility test; Lane agility test; Sprint test.	IMTP peak force and - 1RM squat (*r =* 0.705) - 1RM hang clean (*r =* 0.89) - CMJ height (*r =* 0.809) - Proagility time (*r =* -0.657) - Lane agility time (*r =* -0.523) - Sprint time (*r =* -0.619 to 0.696) - Sprint average velocity (*r =* 0.496 to 0.704) - Sprint peak velocity (*r =* 0.498 to 0.536) - Sprint average force (*r =* 0.482 to 0.685) - Sprint average power (*r =* 0.621 to 0.728). IMTP RFD at various time point vs - 1RM hang clean (*r =* 0.668 to 0.701) - CMJ height (*r =* 0.556 to 0.570) - Sprint time (*r =* -0.432 to 0.472) - Sprint average velocity (*r =* 0.426 to 0.493) - Sprint peak velocity (*r =* 0.438 to 0.497) - Sprint average force (*r =* 0.415 to 0.589) - Sprint average power (*r =* 0.427 to 0.593).
West et al. (2016)	Lower limb	IMTP; 1RM squat; Proagility test; T-test; 10-m sprint.	IMTP peak force vs - 1RM squat (*r =* 0.866). IMTP force at 90-250 ms vs - 1RM squat (*r =* 0.757 – 0.816). IMTP peak and RFD (0-30 to 0-250 ms) vs. - 1RM squat (*r =* 0.595 to 0.748) - proagility test time (*r =* -0.518 to -0.528) - 5-m sprint time (*r =* -0.527 to -0.570).
West et al. (2011)	Lower limb	IMTP; CMJ; 10-m sprint.	IMTP peak force vs. - 10 m sprint time (*r =* -0.23) - CMJ concentric power (*r =* 0.52) IMTP relative peak force vs. - 10 m sprint time (*r =* -0.37), - CMJ height (*r =* 0.45) IMTP RFD vs. - 10 m sprint time (*r =* -0.66) - CMJ height (*r =* 0.39) IMTP force at 100 ms vs. - 10 m sprint time (*r =* -0.54) - CMJ concentric power (*r =* 0.55) IMTP relative force at 100 ms vs. - 10 m sprint time (*r =* -0.68). - CMJ height (*r =* 0.43) - CMJ concentric power (*r =* 0.38)
Wilson et al. (1995)	Lower limb	ISqT at 110° & 150° knee angles; SJ at 110° & 150° knee angles; CMJ; 30-m sprint.	ISqT RFD at 110° & 150° knee angles vs. - SJ RFD at 110° knee angle (*r =* 0.573 & 0.548, respectively).
Young & Bilby (1993)	Lower limb	Vertical jump; 1RM squat; ISqT; anthropometric measures.	ISqT peak force vs. - Absolute 1RM squat (*r =* 0.71). ISqT relative isometric peak force vs. - Vertical jump (*r =* 0.52) - Absolute and relative 1RM squat (*r =* 0.53 & 0.78, respectively).
Young et al. (1995)	Lower limb	ISqT at 120° knee angle; 50-m sprint; CMJ & SJ at 90° knee angle with 9kg load; SJ at 120° knee angle with 19kg load; Drop jump from 30, 45, 60 and 70 cm box.	ISqT peak force vs. - sprint start (*r =* −0.72)
Young et al. (1999)	Lower limb	Standing CMJ; 1, 3, 5 & 7 steps run up jumps; loaded SJ; DJ; ISqT at 120° knee angle.	ISqT relative peak force vs. - CMJ (*r =* 0.33) - Run up jump (*r =* 0.33)

**Table 4:** Relation between isometric test and performance of sports specific movements.

Authors	Type of Sport	Tests Studied	Results
Leary et al. (2012)	Golf	IMTP; SJ; CMJ; Golf swing.	IMTP allometrically scaled force at 150 ms - Golf club head average and maximum speed (*r =* 0.46 & 0.47, respectively)
Loturco et al. (2016)	Boxing	ISqT & bench press; Fixed and self-selected position jab & cross impact measurement.	ISqT peak force vs. - Fixed position jab impact (*r =* 0.68) - Self-selected position jab impact (*r =* 0.69) - Fixed position cross impact (*r =* 0.83) - Self-selected position cross impact (*r =* 0.73)
Stone et al. (2003)	Throwing	IMTP; Dynamic midthigh pull; Shot put; Weight throw.	IMTP peak force vs. - Shot put (*r =* 0.67 to 0.75) - Weight throw (*r =* 0.70 to 0.79)
Stone et al. (2004)	Cycling	Part 1 & 2: IMTP; Wingate test.Part 2 only: Track cycling time	IMTP peak force vs. - Wingate peak power (*r =* 0.74 to 0.90) - Track cycling split times (*r =* −0.49 to −0.55)
Uali et al. (2012)	Kayaking	Isometric bilateral bench pull & one-armed cable row; Kayak sprint test.	Isometric bilateral bench pull peak force vs - Sprint test time to 2, 5 & 10 m (*r =* −0.718, −0.776 & −0.801, respectively) - Peak velocity (*r =* 0.834) Isometric one-armed cable row peak force vs. - Sprint test time to 5 & 10 m (*r =* 0.731 & −0.700, respectively) - Peak velocity (*r =* 0.653)
van Someran & Howatson (2008)	Kayaking	200-m, 500-m & 1000-m kayak time trial; isokinetic and isometric pulling strength and power. performed in a position that simulated kayak stroke.	Isometric peak force vs. - 200-m sprint time (*r =* −0.47) - 500-m sprint time (*r =* −0.60)
van Someran & Palmer (2003)	Kayaking	200-m kayak time trial; isokinetic and isometric pulling strength and power performed in a position that simulated kayak stroke.	Isometric peak force vs. - 200-m sprint time (*r =* 0.37)
Wells et al. (2018)	Golf	IMTP; SJ; CMJ; DJ; Clubhead velocity assessment.	Isometric peak force vs - clubhead velocity (*r =* 0.482), Isometric RFD 0-150 ms vs - clubhead velocity (*r =* 0.343) Isometric RFD 0-200 ms - clubhead velocity (*r =* 0.398)

CMJ–countermovement jump; COD–change of direction; DJ–drop jump; IBP–isometric bench press; IMTP–isometric mid-thigh pull; ISqT–isometric squat; RFD–rate of force development; SJ–squat jump.
